# An examination of variables associated with breast cancer early detection behaviors of women

**DOI:** 10.4314/ahs.v22i3.16

**Published:** 2022-09

**Authors:** Sultan Kayan, Ilgun Ozen Cinar

**Affiliations:** 1 Faculty of Health Science, Public Health Nursing Department, Bilecik Seyh Edebali University, Bilecik, Turkey; 2 Faculty of Health Science, Public Health Nursing Department, Pamukkale University, Denizli, Turkey

**Keywords:** Breast cancer fear, breast cancer knowledge, early detection, mammography

## Abstract

**Background:**

Breast cancer is a worldwide common public health problem, and it is quite important to know the factors preventing the early detection behaviors to fight against it.

**Objective:**

The aim of the study was to examine the effect of some sociodemographic variables associated with women's breast cancer detection behaviors and their breast cancer knowledge and fear levels.

**Methods:**

The cross-sectional study was conducted with 363 women aged 40–69 who had presented to Cancer Early Diagnosis and Education Centers (CEDEC).

**Results:**

The average age of women is 54.8±7.1. The mean score of breast cancer knowledge (CBCKT) was found as 10.72±2.34, and the breast cancer fear score was found as 27.6±6.5. The percentage of women who regularly breast self-examination (BSE) was 17.4%, clinical breast examination (CBE) was 13.5% and mammography was 42.7%. BSE and having a higher education correlated 6.25-fold. A 6.5-fold correlation was found between BSE and having a family history of breast cancer, and a 6.24-fold correlation between BSE and having information about breast cancer. In CBE, the related variables that affected women receiving information 4.42 times and going to CEDEC 5.3 times. It was found that employment (4.58) of women affected the mammography detection behavior mostly. While women's CBCKT score affected BSE behavior 1.16 times, fear of breast cancer was a variable that affected mammography behavior 2.1 times. It was determined that high CBCKT scores of women increased BSE behaviors 1.16 times, and high breast cancer fear scores increased mammography behavior 2.1 times.

**Conclusions:**

Early detection practices of women are not sufficient in our study. An increase in the knowledge level of women and consideration of the variables determined to be effective in early detection behaviors will allow increasing detection behavior.

## Introduction

Breast cancer is an important health problem with high mortality and morbidity rates among females in developed and developing countries.^1^-^2^ While about 2.1 million women are newly diagnosed with breast cancer every year all over the world, breast cancer constitutes 24.2% of all female cancer types.^3^ Breast cancer is one of the most common cancers among women in Turkey and its incidence is 45.6 per hundred thousand.^4^

Early screening to improve breast cancer outcomes and survival rate is the cornerstone of breast cancer control.^2^ Thanks to breast cancer detection methods, 63.7% of breast cancer diagnoses can be made during the early localized stage.^5^ In the related studies, attention is drawn to early detection methods such as mammography, clinical breast examination (CBE) and breast self-examination (BSE).^6^-^7^-^8^-^9^-^10^-^11^ Mammography is the only breast cancer detection method with proven effect. Studies reveal that screening mammography provides an early diagnosis of 80–90% and reduces the mortality rate by 23–49%.^12^-^13^-^14^ CBE is used as a remarkable method due to the fact that it does not require any tools, it is low cost and accessible and it detects about 26% of breast cancers, 5–7% of cancers missed in mammography.^10^-^15^ Even though there is no evidence that BSE is an effective detection method, it is recommended to encourage women to take responsibility for their health and to increase their awareness level.^2^ In the related studies, it is stated that 80% of the masses in the breast are noticed by women themselves and 20% of them are malign.^5^-^15^-^16^-^9^ Cancer Early Diagnosis and Education Centers (CEDEC), established to carry out the cancer control program in Turkey, run these scans free of charge. The recommended detection program; BSE once a month for every woman over the age of 20, once every two years for over 20 years old, CBE once a year for over 40 years old and a biennial mammogram between the ages of 40 and 69.17

It has been shown in studies evaluating women's knowledge levels that they do not have adequate knowledge about breast cancer and early detection behaviors.^18^-^19^-^20^ Studies to measure the breast cancer knowledge level of women show that women with low levels of breast cancer knowledge are less likely to participate in early detection behaviors. 18-19-20 Fear of breast cancer is another factor that negatively affects early detection behaviors.^21^-^22^-^23^ It is quite important to know the fear and inadequate knowledge levels of women at risk of breast cancer which prevent early detection behaviors in terms of planning the interventions to be performed.^24^ In studies revealing that some socio-demographic characteristics are also effective in breast cancer detection behaviors of women, it is stated that age, education level, marital status, income status, employment status and family history of breast cancer are all effective in general.^25^-^26^-^27^-^28^-^29^

Even though there are studies in the literature evaluating factors affecting early detection behaviors, the present study aimed to determine the variables affecting breast cancer knowledge and fear levels and early detection behaviors of women in the 40–69 age group. Although there are studies evaluating the factors affecting the early detection behaviors in the literature, this study aimed to determine the breast cancer early detection behaviors of women aged 40–69 who are at risk for breast cancer, and the variables affecting it, as well as their breast cancer knowledge and fear levels.

## Methods

### Study design and sample

The present study is a cross-sectional study. The universe is made of 174 008 women between the ages of 40–69 living in a city of the country.^30^ The sample formula with a known universe was used so as to determine the sample. 31 According to the calculation, the rate of women with no knowledge of breast cancer, 37.5% 19, the alpha value is 0.05, the t value is 1.96, and the deviation amount (d) is 0.05, and the sample size is 359. Considering the risk of sample loss, more participants were included in the study in order to have a strong sample. While collecting the data, a total of 363 women were included in the study using the random sampling method 32, one of the non-probability sampling methods. The researchers collected data between the dates 21 May and 21 September 2018.

### Study population

The research included women (1) between the ages of 40–69, (2) no history of breast cancer, (3) no communication problems, and (4) who volunteered for the study.

### Assessment tools

The data were collected with the following forms.

### Information Form About Women

The said form consisting of 22 questions prepared in line with the literature 19-33-34-35, comprises questions on socio-demographic characteristics of women (age, marital status, educational status, place of residence) breast cancer and early screening practices (breast cancer history in the family, getting information about breast cancer, performing BSE, having CBE, having mammography, applying to CEDEC).

### The Comprehensive Breast Cancer Knowledge Test (CBCKT)

The scale was developed by Stager (1993). Reliability & Validity was performed by Basak in Turkey (2015). There are 20 information questions in total and it has two dimensions: general information (questions between 1 and 12) and treatability (questions between 13 and 20). The scale evaluates general information on breast cancer and information on the treatability level of breast cancer. The Cronbach Alpha coefficient for the whole scale was found as 0.90. The scale is answered as Correct-Incorrect. Questions answered as correct are evaluated by 1 point while the questions that are answered as incorrect and the questions that are not answered are evaluated by 0 points. There are 8 correct and 12 incorrect statements in the questions. While questions 1, 2, 5, 6, 11, 12, 14, 15, 17, 18, 19 and 20 contain incorrect statements, others include correct statements.^36^-^37^

### Breast cancer fear scale

The Cronbach Alpha coefficient of the scale developed in 2004 by Champion et al. was reported as 0.91.38 It was adapted to Turkish by Secginli and the Cronbach Alpha coefficient of the scale was found as 0.85.39 The scale consisting of eight items determines the correlation between breast cancer, mammography behavior, and emotional responses of women. The scale is scored from 1 to 5; “strongly disagree” (1), “disagree” (2), “undecided” (3), “agree” (4), “strongly agree” (5). The highest score that can be achieved from the scale is 40 and the lowest score is 8. According to the evaluation, 8–15 points indicates low-level fear, 16–23 points moderate fear and 24–40 points high-level fear.^38^-^39^ In the present study, the Cronbach Alpha coefficient of the scale was found as 0.89.

### Data Collection Procedures

There are two CEDECs (Pamukkale and Merkezefendi) in the city where the study to be conducted. The population registered in Pamukkale CEDEC is higher. The women who applied to these centers were interviewed right after the registration before the screening practices and training activities. Data were collected from women who met the inclusion criteria in a quiet environment using the face-to-face interview method. Of the research sample, 61.1% (n=222) was from Pamukkale CEDEC, 38.9% (n=141) was from Merkezefendi CEDEC data. Since the number of applying to Pamukkale CEDEC is higher, more participants from there were included in the sample. 60 women from Pamukkale CEDEC and 25 women from Merkezefendi CEDEC were excluded since they did not want to participate in the study.

### Statistical Analysis

Statistical analysis was done with SPSS 24.0 software.40 The mean, standard deviations are given for descriptive continuous variables while frequency and percentage distributions are given for categorical variables. The compliance of the scales to the normal distribution was evaluated with the Kolmogrow Simirnov test, and the mean scores and standard deviations were also calculated. Significantly related variables on BSE, CBE and mammography behaviors were separately included in the logistic regression (LR) model after the basic analysis (age, education, marital status, employment status, menopause, breast cancer history in the family, getting information, applying to CEDEC, CBCKT and fear scale score averages). Statistical significance value was taken as p<0.05.

## Results

The distribution of women according to their socio-demographic characteristics, medical histories and early detection behaviors are presented in Table 1. It was found that 82.9% of the women had 8 years or less education, 74.7% were married, 74.1% were in menopause and their mean age was 54.8±7.1. The regular BSE rate was 17.4%, the regular CBE rate was 13.5% and the regular mammography rate was 42.7%. It was determined that 52.6% of the women applied to CEDEC for the first time. The total score average of women from CBCKT was 10.72±2.34, while breast cancer fear scores were 27.68±6.50 (Table 2).

**Table 1:** Descriptive characteristics of participants

Demographics and Medical History		Number	%
	40–54	153	42.1
Age	55–69	210	57.9
Mean (SD)		54.8±7.1	
Educational status	8 years & less	301	82.9
	8 years & over	62	17.1
Marital Status	Married	271	74.7
	Single	92	25.3
Employment Status	Unemployed	295	81.3
	Employed	68	18.7
Menopause	Yes	269	74.1
	No	94	25.9
Family history of BC	Yes	82	22.6
	No	281	77.4
BSE	Done regularly (monthly basis)	63	17.4
	Done never or irregularly	300	82.6
CBE	Done regularly (annual basis)	49	13.5
	Done never or irregularly	314	86.5
Mammography	Done regularly (every 2 years)	155	42.7
	Done never or irregularly	208	57.3
Got information about breast	Yes	253	69.7
cancer	No	110	30.3
CEDEC applying	First applying	191	52.6
	Twice and more applying	172	47.3
Total		363	100

**Table 2:** Women's breast cancer knowledge and fear mean scores

	Mean±SD	Median	Min-Max
General Knowledge Sub-Dimension	6.47±1.60	6.00	2–11
Treatability Sub-Dimension	4.26±1.47	4.00	0–8
CBCKT Total Score	10.72±2.34	11.00	5–19
Breast Cancer Fear Score	27.68±6.50	29.00	8–40

In the logistic regression model, the age variable BSE and CBE had an effect increasing the detection behavior about one fold (p<0.01). The fact that women had 8 years or more education was determined as a statistically significant variable affecting BSE behavior 6.25 times (p<0.001), CBE behavior 2.5 times (p<0.01), and mammography behavior 4.07 (p<0.05) times. It was determined that being married 3.81 times (p<0.01), being in menopause 3.64 times (p<0.001), and having a family history of breast cancer were 6.5 times (p<0.001) effective variables in the BSI behavior of women. Women's employment status was found as a statistically significant variable affecting BSE behavior 4.31 times (p<0.001) and CBE behavior 4.58 (p<0.05) times. The fact that women got information on breast cancer affected BSE behavior 6.54 times (p<0.001) and CBE behavior 4.42 (p<0.01) times. The fact that women made more than one application to CEDEC was determined as a statistically significant variable that was 2.21 times (p<0.01) in BSE behavior, 5.30 times (p<0.001) in CBE behavior, and 2.10 (p<0.05) times in mammography behavior. CBCKT score, BSE behavior 1.16 times (p<0.01), breast cancer fear score was found as statistically significant variables affecting mammography behavior of women 1.05 (p<0.05) times (Table 3).

**Table 3:** The factors affecting early detection behaviors according to the LR model

Variables	BSE			CBE			Mammography		
	p	OR	(95%CI)	p	OR	(95%CI)	p	OR	(95%CI)
**Age (Mean)**	**0.000**	0.90	0.86–0.94	**0.019**	0.95	0.91–0.99	0.298	0.97	0.92–1.02
**Education (1)** 8 years & less: 0 8 years & over: 1	**0.000**	6.25	3.39–11.54	**0.008**	2.50	1.26–4.95	**0.048**	4.07	0.95–17.39
**Marital Status** **(1)** Married: 1 Single: 0	**0.003**	3.81	1.58–9.18	0.617	1.20	0.58–2.46	0.303	0.63	0.27–1.50
**Employment** **Status (1)** Unemployed: 0 Employed: 1	**0.000**	4.31	2.37–7.85	0.269	1.49	0.73–3.04	**0.039**	4.58	1.07–19.54
**Menopause (1)** Yes: 0 No: 1	**0.000**	3.64	2.06–6.42	0.646	1.17	0.59–2.28	0.399	0.73	0.35–1.51
**Family history** **of BC (1)** Yes: 1 No: 0	**0.006**	6.50	1.72–14.52	0.263	0.63	0.28–1.41	0.865	0.93	0.42–2.06
**Got** **information** **about BC (1)** Yes: 1 No: 0	**0.000**	6.24	2.43–16.05	**0.002**	4.42	1.70–11.48	0.098	1.78	0.89–3.55
**CEDEC** **applying (1)** Once: 0 Twice and more: 1	**0.006**	2.21	1.26–3.88	**0.000**	5.30	2.55–11.01	**0.043**	2.10	1.02–4.30
**CBCKT**	**0.013**	1.16	1.03–.130	0.143	0.90	0.79–1.03	0.101	1.13	0.97–1.31
**Fear Scale**	0.545	1.01	0.97–1.06	0.052	0.95	0.91–1.00	**0.040**	1.05	1.00–1.11

## Discussion

In the present study, women had low rates of regular BSE and CBE, while rates of mammography in the last two years were moderate (Table 1). While the women's total CBCKT score was determined at a moderate level, the fear of breast cancer was found to be high (Table 2).

According to Eurostat (2017) data, the rate of women showing early detection behavior varies by 20–80%, this ratio is high in developed countries and low in developing countries.^41^ In another study carried out in low and middle-income countries, early detection behaviors were determined as 2.2%.42 Studies have revealed that performing BSE varies by 9–36%, CBE by 4–26% and mammography by 5–39%.^43^-^44^-^45^-^46^-^47^-^48^-^49^ Even though breast cancer is the most common, deadly and preventable problem, the early detection behaviors and implementation efforts of women are still inadequate.

When the factors affecting the BSE behavior of the participants are examined with LR, women's age, education status, marital status, employment status, menopausal status, family history of breast cancer, getting information about breast cancer and previous application to CEDEC were found associated. Even though the evidence of BSE in terms of breast cancer mortality in developed countries is insufficient, it is suggested in developing countries to increase awareness of breast cancer.^5^-^9^-^15^ In the present study, the age variable associated with BSE was found in other studies, and it was emphasized that women in the younger age group perform BSE more frequently.^51^-^52^-^53^-^54^-^55^ It is observed that there is a positive correlation between education level and BSE. Women with higher levels of education perform BSE more frequently. Results are similar in studies of the literature.^27^-^54^-^55^-^56^-^57^-^58^-^59^-^60^-^61^ It is reported that breast cancer perception and health beliefs of women with a higher level of education have a positive correlation.^62^-^63^ In our study, when the marital status variable with BSE is evaluated following the LR model, it was found that married women performed more BSE behavior. It is further emphasized in the studies of the literature that married women perform BSE more frequently.^51^-^54^-^55^-^59^-^64^-^65^-^66^ The social support that married women receive from their spouses and children has a positive effect on detection behaviors.^67^-^68^ The employment status of women, which we specified as another variable that affects BSE, has a positive effect on studies in the literature.^49^-^52^-^55^-^58^-^69^-^70^-^71^-^72^-^73^ Women's education status, marital sttus and employment status are modifiable factors in terms of increasing early diagnosis behaviors and they affect women's life positively.

In the present study, a correlation was found between performing BSE and the fact that women have not gone through menopause. Yilmazel (2013) found that women who have not gone through menopause are more likely to perform BSE than those who have already in menopause.^74^ The fact that women who are not in the menopause period are in the younger age group may be effective in this. In the present study, it was determined that having a family history of breast cancer is also associated with BSE. Different studies have further revealed that family history is effective in BSE behavior.^52^-^69^-^72^-^73^-^75^-^76^ The fact that women with a family history see themselves at risk for breast cancer and know that they are prone to have breast cancer makes it more likely for them to perform BSE more regularly.

It is further emphasized by studies 53-55-79-80 conducted in Turkey 77-78 and other countries that women who know about breast cancer perform BSE more frequently. Further, in the present study, it was determined that getting information about breast cancer increased BSE and CBE but did not affect mammography behavior. Hajian Tilaki and Auladi^55^ further emphasize that getting information about breast cancer increases CBE.Even though one-third of the participants stated that they did not receive any information, the CBCKT total score of the women in our study was determined to be moderate. İnformation points in two studies conducted on the same scale in Turkey were detected to be moderate.^65^-^81^ In other studies in which women's breast cancer knowledge levels were evaluated with different scales, it was stated that their knowledge level was moderate.^34^-^82^-^83^-^84^-^85^-^86^-^87^ In two studies conducted on young women in Ethiopia, the breast cancer knowledge level of women was found as low.^88^-^89^ Remarkably, the studies in which the knowledge level of breast cancer was determined as low and moderate level was in developing or underdeveloped countries.^86^-^87^-^88^-^89^

Due to the fact that increasing the awareness level of breast cancer among women will increase early detection behaviors, it is an issue that should be emphasized. It was found in the present study that the knowledge level of women increased their rate of doing BSI 1.16 times. Studies in the literature further reveal this correlation between knowledge level and BSE behavior.^50^-^69^-^86^-^90^ It is essential for women to get information about breast cancer, to participate in early detection practices by transforming the information they receive into behavior and perform it regularly. Women should be informed in a planned and continuous manner bhealthcare personnel.

We determined a correlation between women's previous application to CEDEC and their BSE, CBE and mammography. This situation reveals that the services provided in diagnostic centers are significant. Women who apply to CEDEC are informed about early screening behaviors and detection practices are performed for women in Turkey. It is quite essential to spread such centers and to make sure that they provide continuous service.

Even though fewer studies are examining the effect of CBE on breast cancer mortality, it is known that it is important in terms of the diagnosis of masses that cannot be visualized by mammography or that do not fall within the limits of mammography in women with dense breast tissue.10–15 In the present study, the age variable affects about one-fold the behavior of having CBE. Other studies in the literature reveal that the younger age group of women raises the CBE rates.^52^-^64^-^65^-^77^ In our study, a correlation was found between the education level of women and their CBE application. As women's educational level increases, CBE behavior increases.^54^-^57^-^91^ It is further considered that increasing the education level of women also increases their knowledge and awareness of breast cancer.

When the factors affecting the participants' having mammography are examined with LR, it was detected that women's educational status, employment status, getting information about breast cancer and applying to CEDEC before were all related. In the present study, when the age variable was considered together with other variables, it was not effective on mammography. In the studies, it has been reported that women in the age group above the age of 40 usually perform more mammography behavior.^51^-^65^-^92^ In the studies in the literature, it was revealed that women with higher education level perform higher mammography behavior.^24^-^54^-^57^-^93^ It was further revealed that unemployed women have more mammograms than employed ones, and part-time workers have more mammograms than full-time workers.^92^ Women with a higher level of education have more opportunities to find a job. For this reason, these two are variables that positively affect women's early detection behavior and require effort from women.

While fear of breast cancer was found high in our study and fear of breast cancer in women was found related to mammography behavior in LR analysis, BSE and CBE were not found related to detection behaviors. Generally, fear of breast cancer was found high in studies.^29^-^81^-^91^-^94^ Studies are revealing the correlation between breast cancer fear and having mammography 95 and BSE behavior.^91^ There are also some studies revealing that the fear of breast cancer is not effective on mammography behavior^29^-^96^ and that it is an obstacle to perform BSE behavior.^23^ Tuzcu et al.^97^ stated that the fear of breast cancer is both a motivating and a hindering factor in detection behavior. Individuals may present compatible or incompatible responses to fear. Regarding the fear of breast cancer, it can be assumed that women's approaches to early detection behaviors are individual. While evaluating women's early detection behaviors, variables determined to be effective should be taken into consideration.

## Conclusion

Early detection behavior practices of women are not at a sufficient level. In this study, the effective variables of women's breast cancer detection behaviors were evaluated. The high education level of women in BSE, CBE, and mammography behaviors and their previous application to CEDEC were determined as common effective variables. Factors affecting BSE behavior include age, education, being married or single, employment, menopause, family history of cancer, and information retrieval variables. Fear of breast cancer was found effective only on having a mammography.

It is essential to inform women with regular breast cancer education programs, to turn the repetitive information given into behavior and to ensure early detection behaviors. CEDECs, which are effective in screening behaviors of women, are quite noteworthy centers in terms of both screening and informing them. Women should be informed about these centers and it should be ensured that they use these centers effectively. All healthcare professionals should provide training on this subject in the field they work.

## List of abbreviations

BC: Breast Cancer
BSE: Breast Self-Examination
CBE: Clinical Breast Examination
CEDEC: Cancer Early Diagnosis and Education Centers
SD: Standard deviation
CBCKT: Comprehensive Breast Cancer Knowledge Test
Cl: Confidence interval, OR: Odds ratio

## Limitations

The data includes women who applied to CEDEC units of a city in the country.
